# Sanziguben polysaccharides inhibit diabetic nephropathy through NF-κB-mediated anti-inflammation

**DOI:** 10.1186/s12986-021-00601-z

**Published:** 2021-09-07

**Authors:** Kang Zhou, Jianing Zhang, Chang Liu, Lijuan Ou, Fan Wang, Yang Yu, Yumei Wang, Shasha Bai

**Affiliations:** School of Pharmaceutical Sciences, Guangzhou University of Chinese Medicine, Room C306, Pharmaceutical Building, No. 232 Waihuan East Road, Panyu District, Guangzhou, Guangdong China

**Keywords:** Sanziguben polysaccharides, Diabetes nephropathy, Inflammation

## Abstract

**Background:**

Sanziguben polysaccharides (SZP) are large amounts of classical Chinese medicines from Sanziguben (SZGB). Moreover, SZGB is a widely applied compound prescription for diabetic nephropathy (DN) treatment, but the role is still unclear. This study initially explores the mechanism of SZP in the treatment of DN.

**Methods:**

The high-fat diet plus streptozotocin injections were used to replicate the DN models in male C57BL/6 mice. DN mice were divided into five groups: DN mice, DN mice treated with SZP(1.01 or 2.02 g/kg), DN mice treated with SZGB decoction(4.7 g/kg), and DN mice treated with metformin (300 mg/kg). HG and LPS plus TNFα stimulated human tubule epithelial (HK-2) cells to establish an in vitro model and treated with SZP (100 or 200 μg/mL).

**Results:**

SZP was found to comprise sugar, protein, and uronic acid. Furthermore, SZP alleviated the progression of inflammation in vivo and in vitro by inhibiting the expression of NF-κB.

**Conclusions:**

NF-κB plays a critical role in the development of DN induced by STZ and HG. Furthermore, SZP can attenuate the NF-κB‐mediated progression of diabetic nephropathy, improve DN through anti-inflammation.

## Introduction

Diabetic nephropathy (DN) is the most common diabetic microvascular complication majorly affecting the quality of human health worldwide [1,2]. The mechanisms of DN pathogenesis are still not fully elucidated despite significant insights into DN in recent decades [3]. Blood glucose, the parameters of renal function, and histopathology have been identified as the main clinical characteristics of DN [4]. Moreover, inflammation and immune response are likely to be closely associated with the occurrence and development of DN. Clinical and experimental studies have demonstrated that excessive inflammation can lead to immune damage in the body, causing a variety of diseases and DN [1]. A study reported that monocytes from type 2 diabetes mellitus (T2DM) patients had a proinflammatory profile with a marked capacity for the expression of inflammatory cytokines. Nuclear factor-kappa B (NF-κB) significantly promoted the development of inflammation in kidney tissues. Several studies have shown the effects of nuclear factor NF-κB p65 expression in monocytes from patients with T2DM and DN with uremia [5].

Metformin (MET) could promote pancreatic β-cell functions and decrease hepatic glucose production and intestinal glucose absorption to protect the kidney. Multiple natural compounds with various biological activities have become a treasure trove for researchers developing new drugs. Consequently, many groups have successfully confirmed that polysaccharides could regulate oxidative stress and inflammatory factors [6–8].

Sanziguben is a compound prescription made from four kinds of Chinese herbs: *Gynostemma pentaphyllum* (Thunb.) Makino, Chinese *Rosa laevigata* Michx, *Schisandra chinensis* Fructus, and *Phyllanthus emblica* and Fructus. A previous study has demonstrated that these herbs have anti-inflammatory [9] and antidiabetic [10] effects and are widely used for DN prevention [4]. Studies have shown that SZGB contains a high content of polysaccharides. Moreover, Sanziguben polysaccharide (SZP) is a polysaccharide from SZGB. Therefore, Sanziguben polysaccharide (SZP) is proposed to improve diabetic nephropathy through anti-inflammation.

This study used the high-fat diet (HFD) plus streptozotocin (STZ)-induced mice and high glucose (HG)and LPS plus TNFα stimulated human tubule epithelial (HK-2) cell models to assess the anti-inflammatory effects of SZP and find its possible molecular mechanism.

## Materials and methods

### Plant materials

Sanziguben (SZGB) is composed of four Chinese herbs: *G. pentaphyllum* (Thunb.) Makino, Chinese *R. laevigata* Michx, *S. chinensis* Fructus, and *P. emblica* and Fructus. The crushed herbs of Schizandrae were purchased from Daxiang Pharmaceuticals Inc. (Guangzhou, China).

### Chemicals

The reference standard of glucose and uronic acid were provided by Shanghai Yuanye Biotechnology Co., Ltd. (Shanghai, China). Moreover, metformin hydrochloride tablets were purchased from Sino-American Shanghai Squibb Pharmaceuticals Ltd. (Shanghai, China). STZ was obtained from Sigma-Aldrich (Sacramento, CA, USA). All test assay kits and enzyme-linked immunosorbent assay (ELISA) kits were supplied by NanJing JianCheng Bioengineering Institute (Nanjing, China). Tumor necrosis factor-alpha (TNF-α) was obtained from PeproTech Inc. (Cranbury, NJ, USA). Lipopolysaccharides (LPS) were provided by Beijing Solarbio Science & Technology Co., Ltd. (Beijing, China). NF-κB p65 (1:2,000), phospho-NF-κB p65(Ser536; 1:2,000), and phospho-IκBα (Ser32; 1:2,000) were purchased from Cell Signaling Technology (Beverly, MA, USA). The radioimmunoprecipitation assay (RIPA) lysis buffer with protease/phosphatase inhibitor cocktail, bicinchoninic acid (BCA) protein assay, anti-β-actin antibodies (1:2,000), and horseradish peroxidase (HRP)-conjugated secondary antibodies was purchased from Beijing ComWin Biotech Co., Ltd. (Beijing, China). All other chemical reagents were of analytical grade.

### Extraction

Dried SZBG herbs were pulverized and screened through a 50-mesh sieve. The powder was defatted with petroleum ether for 24 h at room temperature under reflux to remove some colored materials and oligosaccharides. The residue was extracted with distilled water at 80 °C twice and 2.5 h for each time after filtration. The whole extract was filtered and concentrated in a rotary evaporator under reduced pressure at 60 °C for fivefold and then centrifuged at 3,000 rpm for 15 min. Moreover, the extract was then precipitated by the addition of four volumes of 95% ethanol at 4 °C overnight. Moreover, the polysaccharide was obtained by centrifugation (4,000 rpm for 10 min). The solution was reprecipitated by the addition of 95% ethanol as described above, and the resultant precipitate was successively washed with anhydrous ethanol and then dried under reduced pressure to obtain SZP.

### Animals

Male C57BL/6 mice (4 weeks old, 13–15 g) were purchased from the Experimental Animal Center of Guangzhou University of Chinese Medicine (Guangdong, China). Mice were housed in a room with a temperature of 23 °C ± 1 °C and 55% ± 5% relative humidity with a 12-h light/12-h dark cycle. All animal experiments were approved by the Institutional Animal Care and Use Committee of Guangzhou University of Chinese Medicine (license no: SCXK 2013–0020). All animal welfare in this study followed the guidelines for ethical review of animal welfare in the People’s Republic of China.

### Induction of mice and drug administration

The HFD plus STZ injections were used to replicate the diabetic models in mice [11,12]. MET is the first-line drug for the treatment of diabetes and DN [13]. Therefore, MET is selected as a positive control drug. Moreover, the mice were randomly divided into the diabetic and sham groups 1 week after adjusting to the new environment. Mice were fed with either HFD (45% of calories from fat) or standard chow diet (10% of calories from fat) for 10 weeks. Mice in the diabetic group were fed with HFD to accelerate the development of diabetic kidney disease. The diabetic group was intraperitoneally injected with 100 mg/kg STZ dissolved in citrate buffer (pH 4.0). Consequently, mice appeared hyperglycemic with fasting blood glucose (FBG) levels of 11.1–33.3 mmol/L 3 days later. The normal control group was the group that was injected with the citrate buffer without STZ. The following groups were generated for this current study: (1) Sham:normal control mice treated with distilled water, (2) DN:diabetic mice treated with distilled water, (3) SZP-L:diabetic mice treated with SZP (1.01 g/kg body weight/day), (4) SZP-H:diabetic mice treated with SZP (2.02 g/kg body weight/day), (5) SZGB:diabetic mice treated with SZGB (4.7 g/kg body weight/day), and (6)MET: diabetic treated with metformin (300 mg/kg body weight/day). Six mice in each experimental group, and all mice were treated once a day for 8 weeks.

All mice were euthanized at 8 weeks after diabetes induction, and blood samples were collected and centrifuged (3,500 rpm for 10 min) to obtain serum samples. Moreover, the kidneys were removed. The renal cortex of the kidney was isolated immediately and stored in liquid nitrogen for pathological and molecular studies.

### Cultivated HG stimulates HK-2 cells

The HK-2 cells were purchased from China Center for Type Culture Collection (Wuhan, China) and grown in Dulbecco’s modified eagle’s medium/nutrient mixture F-12 (DMEM-F12, 1:1; GIBCO, Life Technologies, Carlsbad, CA, USA), which contained 10% fetal bovine serum (GIBCO) and 1% antibiotic–antimycotic solution (GIBCO). Cells were cultured at 37℃ in a humidified atmosphere with 5% CO_2_. According to the different experiments, different stimuli were used in this study for (1) the cells to be stimulated with normal glucose as normal control, (2) for HG treatment (a final concentration of 90 mmol/L in culture medium), and (3) SZP (a final concentration of 100 or 200 μg/mL in culture medium). Moreover, all cell experiments were repeated thrice. Cell proliferation and morphology were measured using the IncuCyte ZOOM System (Essen, BioScience Inc., Ann Arbor, MI, USA).

### Cultivated LPS + TNF-α stimulates HK-2 cells

HK-2 Cells were cultured at 37℃ in a humidified atmosphere with 5% CO_2_ and were subcultured at 80% confluence using 0.25% trypsin–EDTA solution. The cells were exposed to DMEM-F12 containing 30 ng/mL TNF-α and 30 μg/mL LPS for 16 h to induce an inflammatory milieu. The experiments were divided into the following groups: (1) cells grown in DMEM-F12 complete medium as normal control, (2) cells stimulated by TNF-α (30 ng/mL) and LPS (30 μg/mL) for 16 h to produce inflammatory damage as a model, and (3) 100 or 200 μg/mL SZP simultaneous treatment on the model group. All cell experiments were repeated thrice. Cell proliferation and morphology were measured using the IncuCyte ZOOM System.

### Chemical components of SZP

Carbohydrate contents were measured by the phenol–sulfuric acid colorimetric method with glucose as equivalents. Protein contents were measured by Coomassie brilliant blue reaction using bovine serum albumin as the standard. Uronic acid content was determined by the carbazole-sulfuric acid method with glucuronic acid as the standard [14].

### Monosaccharide composition analysis

The SZP monosaccharide compositions were analyzed by gas chromatography (GC) using an Agilent GC-6890A-5975C system equipped with a Hypercarb column. After trifluoroacetate at 121 °C for hydrolysis with 2 mol/L trifluoroacetate at 121 °C detected with a flame ionization detector (240 °C), the column temperature was increased from 170 °C for 2 min and 240 °C with 6 °C/min holding for 60 min. Moreover, the conversion of hydrolysate into distilled water was used as previously described [15].

### Infrared spectroscopic analysis of polysaccharides

SZP was characterized by Fourier transform infrared spectroscopy (FTIR) on a Thermo Nicolet iS 5N infrared spectrophotometer (Thermo Fisher Scientific, Waltham, MA, USA) at 25 °C using KBr pellets. Samples were dried at 60 °C in a vacuum drying oven for 48 h before analysis, and spectra were scanned between 4000 and 400 cm^−1^ with a resolution of 4 cm^−1^.

### Measurement of body weight, blood glucose, and urinary protein

The bodyweight of mice was measured at 2-week intervals. Blood glucose was detected using the Roche Dynamic Blood Glucose Monitoring System (Roche Inc., Mannheim, Germany) by blood sampling from the tail vein. Mice were kept separately in metabolic cages for 24-h urinary collection. Urinary protein was evaluated using the Bradford method.

### Biochemical analysis

At the end of the experiment, blood samples and cell supernatants were centrifuged at 3000 × *g* for 10 min. Blood samples were separated for the detection of total cholesterol (TC), triglycerides (TG), serum creatinine (Cr) and serum urea (BUN), malondialdehyde (MDA), and catalase (CAT). The levels of the aforementioned biochemical indicators were measured using commercially available kits (Jian Cheng Bioengineering Institute, Nanjing, China). Meanwhile, the serum levels of TNF-α, monocyte chemotactic protein-1 (MCP-1), and interleukin 6 (IL-6) were measured using ELISA kits (Jian Cheng Bioengineering Institute).

### Histopathological analysis

The sections of kidney tissues were removed and immediately fixed in 4% paraformaldehyde, dehydrated through a graded alcohol series, and embedded in paraffin. Moreover, 4-µm thick sections were cut using a rotary microtome and stained with hematoxylin & eosin (H&E staining), periodic acid-Schiff (PAS), and Masson’s trichrome to evaluate the pathological changes of the kidney tissue. The stained specimens inspected were placed under a light microscope (Nikon, Tokyo, Japan) and imaged (× 400).

### Western blot analysis of NF-κB protein expression in Renal tissues or HK-2 cells

Total protein from renal tissues or HK-2 cells was extracted by RIPA lysis buffer with protease/phosphatase inhibitor cocktail and centrifuged at 14,000 × g for 10 min at 4 °C. The BCA protein assay was performed to determine the protein concentration in the supernatants according to the manufacturer’s instructions. An aliquot of the supernatant was then suspended in a 4 × loading buffer. 100 °C metal bath for 5 min to denature the protein. 40 μg or 20 μg of the protein samples in each group electrophoresed on 12% sodium dodecyl sulfate–polyacrylamide gel electrophoresis (SDS-PAGE) gels at 120 V for 90 min, and then transferred onto polyvinylidene difluoride (PVDF) membranes by utilizing a Trans-Blot transfer cell (Bio-Rad, Hercules, CA, USA) at 300 mA for 100 min. After transferring, the PVDF membrane was blocked with 5% skim milk under room temperature for 2 h.The primary antibodies were as follows: NF-κB p65 (1:2,000), phospho-NF-κB p65 (Ser536; 1:2,000), phospho-IκBα (Ser32; 1:2,000), and β-actin (1:2,000). The membrane was stained with the HRP-conjugated secondary antibodies (Beijing ComWin Biotech Co., Ltd. Beijing, China) or fluorescent secondary antibody (IRDye 800CW Goat Anti-Rabbit or IRDye 800CW Goat Anti-Mouse was purchased from LI-COR, Inc., Lincoln, NE, USA) for 1 h at room temperature after being incubated with the primary antibody at 4 °C overnight. The bands were presented by reacting with a chemiluminescent HRP substrate (WBKLS0100, Millipore, Germany) or scanned with odyssey near-infrared two-color laser imaging system(Odyssey SA). The intensities of individual bands were quantified with densitometric analysis using ImageJ (National Institutes of Health, Bethesda, MD, USA), and β-actin expression was considered as the internal reference.

### Statistical analysis

The diagrams and graphs were analyzed using GraphPad Prism 8.0 software and Image J. Data are presented as mean ± standard deviation and analyzed using the Statistical Package for the Social Science, version 20.0 (SPSS 20.0, Armonk, NY, USA). Statistical significance was determined using one-way analysis of variance (ANOVA) followed by LSD test, with p < 0.05 being statistically significant.

## Results

### Physicochemical properties of SZP

The yields of the polysaccharides extracted from defatted SZBG medicines through the hot-water extraction method and subjected to the physiochemical analyses weighed 6.22%. Conversely, SZP was a dark brown powder. The composition of SZP is summarized in Table 1. The sugar and protein contents of SZP were 54.97% and 44.17%, respectively.

**Table 1:** Physicochemical properties of SZP

Sample	Total sugar (%)	Uronic acid (%)	Protein (%)
SZP	41.6 ± 1.49	12.4 ± 3.8	44.17 ± 1.1

Data are presented as the mean ± SD, n = 3

### Monosaccharide composition analysis

The monosaccharide composition of SZP was identified by comparing the relative retention time with their respective standard as well as by comparing their mass fragmentation patterns. The monosaccharide compositions of SZP are shown in Table 2. Gas chromatography–mass spectrometry analysis revealed that SZP contains mainly fucose (0.38%), arabinose (13.23%), galactose (9.75%), glucose (60.88%), xylose (1.34%), mannose (6.82%), galacturonic acid (5.54%), and glucuronic acid (2.06%).

**Table 2:** **.**Monosaccharide composition analysis of SZP

Sample	Monosaccharide composition (molar ration %)							
	Fuc	Ara	Gal	Glc	Xyl	Man	Gal-AC	Glc-AC
SZP	0.38	13.23	9.75	60.88	1.34	6.82	5.54	2.06

### FTIR analysis

**Fig. 1:**
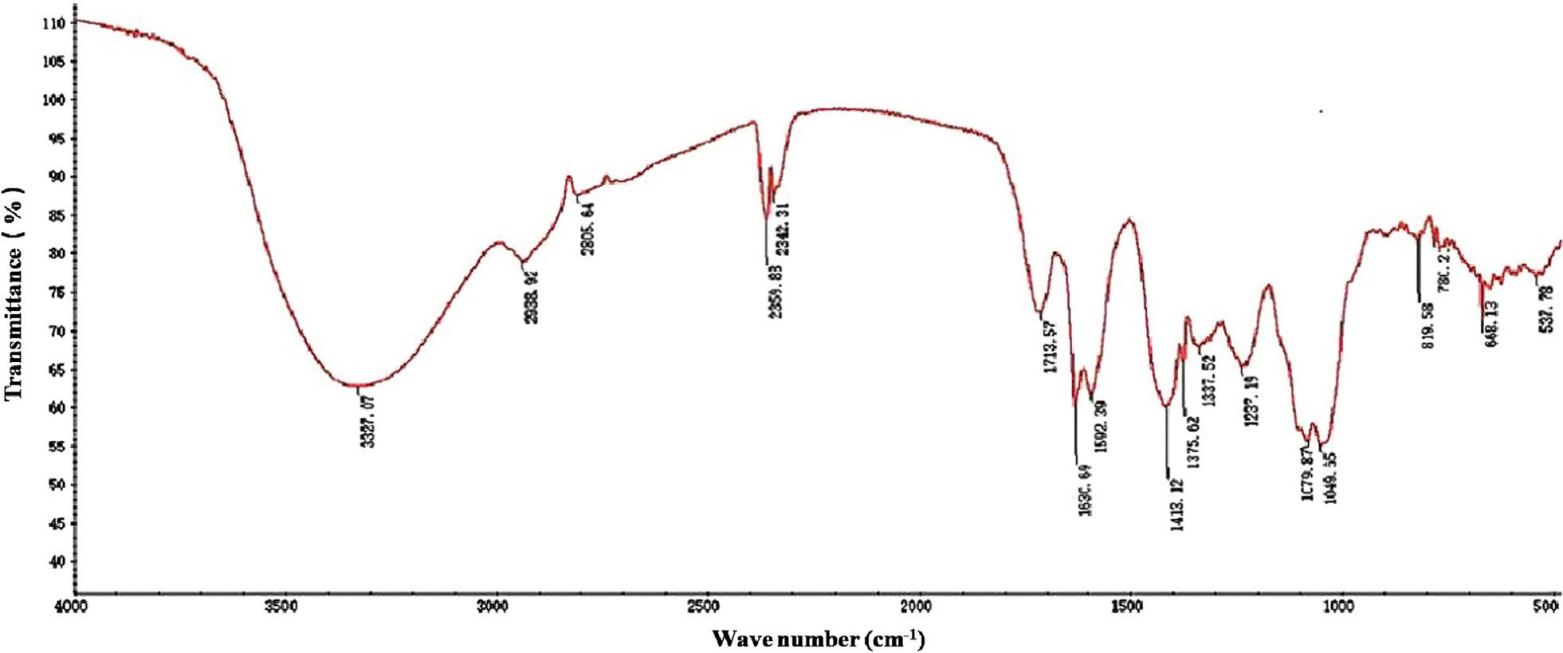
Fourier-transform infrared spectroscopy spectra of SZP

FTIR spectroscopy identified different functional groups, especially O–H, N–H, and C=O. The FTIR spectra of SZP are shown in Fig. 1. The strong and broad absorption peak at 3327 cm^−1^ is attributed to the O–H stretching vibration. A weak absorbance band at around 2938 and 2805 cm^−1^ is a characteristic of polysaccharides, which is due to the C–H stretching and bending vibrations [16]. The strong absorption peak at 1713 and 1630 cm^−1^ indicates the C=O stretching vibration and the carboxyl group. The peak at 1419 cm^−1^ results from the variable angle vibration of the N–H group, which indicated the existence of proteins. The peaks at 1038 and 1120 cm^−1^ belong to the C–O stretching vibration of the pyran ring. The band at 778 cm^−1^ indicated the pyran configurations of polysaccharides [16,17].

### SZP ameliorates symptoms of STZ-induced DN in mice

**Fig. 2:**
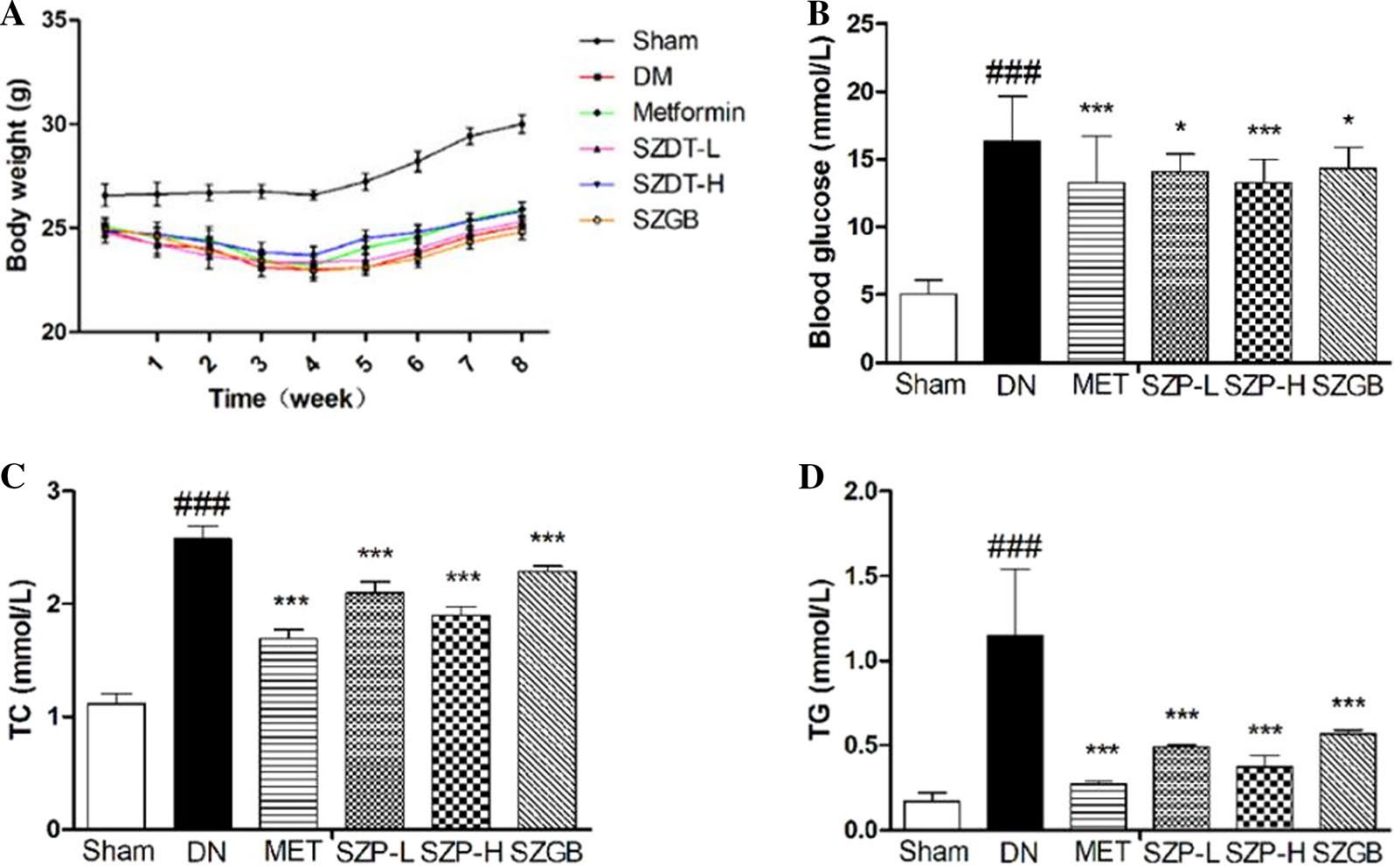
The effects of SZP on the parameters of body weight (**a**), blood glucose (**b**), blood lipids TC (**c**) and TG (**d**) in DN mice. The values presented are the means ± SD of six independent experiments (n = 6) and differences between mean values were assessed by ANOVA. ###P < 0.01 versus sham control group, ***P < 0.01 versus DN group and *P < 0.05 versus DN group. TC, Total cholesterol; TG, triglycerides

The HFD plus STZ-induced DN model was used in the study to evaluate the effects of SZP. The body weight and blood glucose were observed and recorded during the experiment. The food intake of the DN mice was normal, and the body weights of the sham control group mice gradually increased. However, the mice of the DN group showed weight loss, increased food intake, and reduced exercise after STZ administration. The symptoms of mice were markedly ameliorated by adding 2.02 g/kg d dose SZP. The body weight of the SZP-H group increased compared with that of the DN group (Fig. 2a). In addition, the blood glucose of DN mice was significantly higher than that of the sham control. Furthermore, the treatment with SZP did not reverse the levels of blood glucose in these mice as shown in Fig. 2b. SZP markedly decreased the levels of blood glucose when compared with the DN group. HFD plus STZ treatment results in serious DN and leads to various blood lipid abnormalities. The obvious characteristics include the significantly higher levels of TC and TG. Moreover, the contents of TC and TG in serum were analyzed at the end of the experiment. The levels of TC and TG in the DN group were significantly higher than those in the sham control group, and the SZP supplement reduced TC and TG (Fig. 2c, d).

### Effects of SZP on the parameters of renal function in STZ-induced mice

**Fig. 3:**
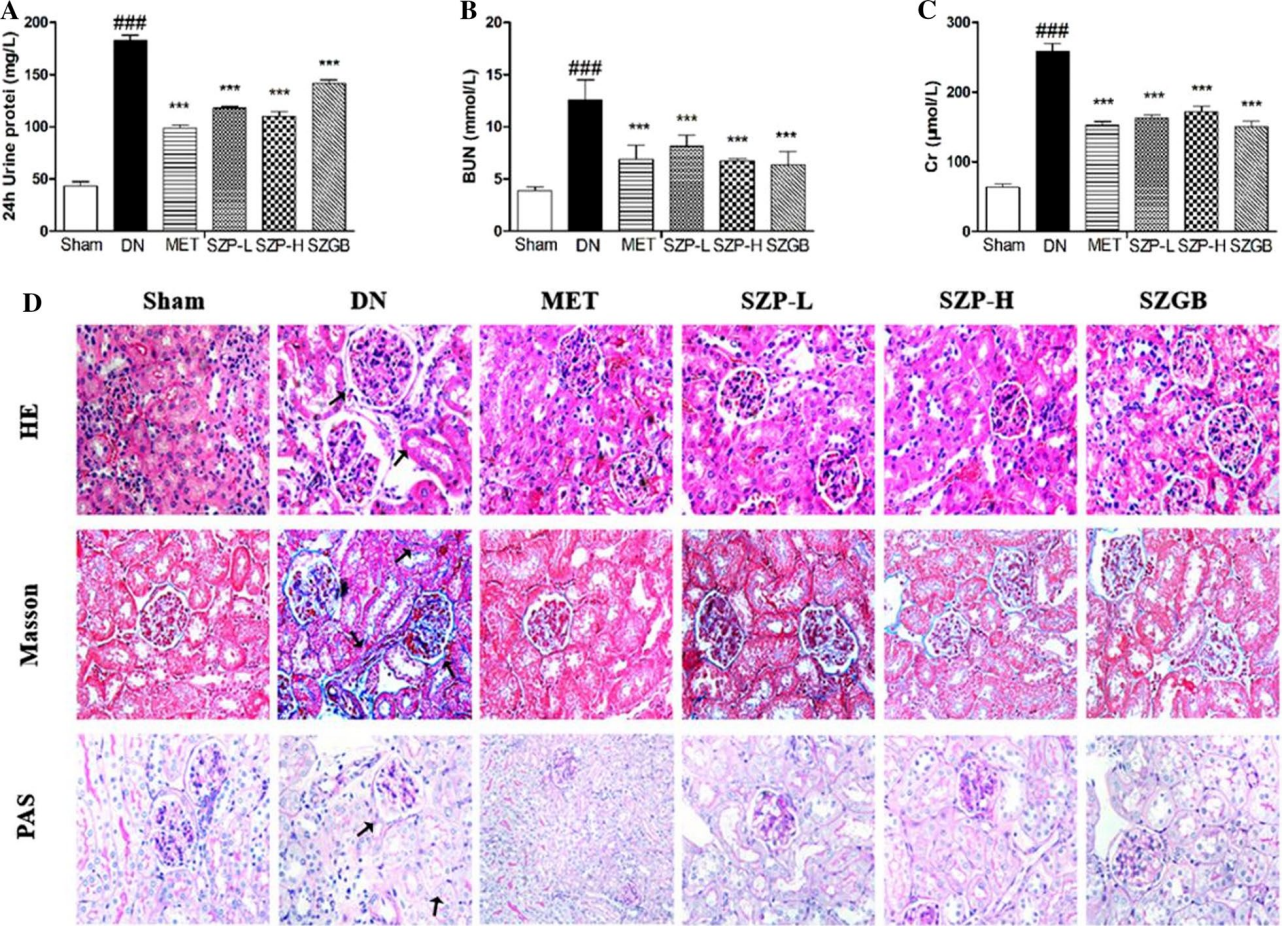
The effects of SZP on the parameters of renal function and kidney tissue in DN mice. The expression level of 24-h urinary protein (**a**). The expression level of serum BUN (**b**). The expression level of Cr (**c**). The Photomicrographs (HE, magnification × 400; PAS, magnification × 400; Masson's trichrome-stained, magnification × 400) of different groups (**d**). Black arrows indicated glomerular hypertrophy, mesangial expansion,tubular dilatation and collagen fiber deposition.The values presented are the means ± SD of six independent experiments (n = 6) and differences between mean values were assessed by ANOVA. ###P < 0.01 versus sham control group, ***P < 0.01 versus DN group. Cr, serum creatinine. HE, hematoxylin and eosin; PAS, periodic acid-Schiff

This study tested the levels of 24-h urinary protein, serum BUN, and Cr to detect the protective effect of SZP on the renal function of DN mice. After administration, compared with the sham control group, The 24-h urine protein (Fig. 3a), serum BUN (Fig. 3b) and Cr (Fig. 3c) of the DN group all showed significant increase. However, they were significantly decreased in the SZP group when compared to the DN group.These indicated that SZP treatment improved the kidney damage in DN mice after modeling.

### Effect of SZP on histopathological kidney changes

Histopathology changes reflect the extent of destruction and inflammation, which are obvious characteristics of DN. The hallmark includes glomerular and tubule–interstitial alterations in the kidney tissues. In this experiment, the kidney tissues were taken for H&E, PAS, and Masson’s trichrome inspection. Compared with the sham control group, H&E and PAS staining showed that the DN group had obvious inflammatory damage such as glomerular hypertrophy, mesangial expansion and tubular dilatation (Which marked by the black arrow). SZP treatment significantly improved the above-mentioned pathological conditions. Masson staining showed glomerular and renal interstitial fibrosis in the DN group (Which marked by the black arrow). SZP treatment improved the above pathological changes caused by the excessive deposition of collagen fibers (Fig. 3d).

### SZP suppressed the expressions of inflammatory cytokines in DN tissues

**Fig. 4:**
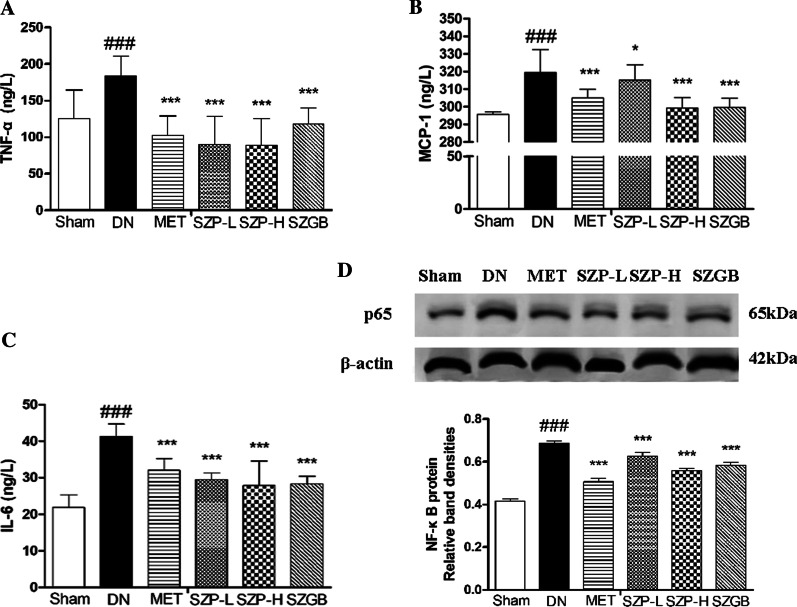
The effects of SZP on the secretion of inflammatory cytokines and proteins in DN mice. The expression level of TNF-α (**a**). The expression level of MCP-1 (**b**). The expression level of IL-6 (**c**). The values presented are the means ± SD of six independent experiments (n = 6) and differences between mean values were assessed by ANOVA. NF-κB protein expressions in DN mice after SZP treatment (**d**). The values presented are the means ± SD of three independent experiments (n = 3) and differences between mean values were assessed by ANOVA. ###P < 0.01 versus sham control group, ***P < 0.01 versus DN group and *P < 0.05 versus DN group. TNF-α, Tumor necrosis factor-α; MCP-1, Monocyte chemotactic protein-1; IL-6, Interleukin 6. NF-κB, Nuclear factor-kappa B

Overexpression of inflammatory cytokines, for example, TNF-α, IL-6, and MCP-1 mediators, plays a crucial role by inhibiting systemic lymphoid function and activated macrophages in DN pathogenesis. The data of this study showed that the inflammatory cytokines TNF-α (Fig. 4a), IL-6 (Fig. 4b) and MCP-1 (Fig. 4c) in the DN group were significantly higher than those in the sham control group. In addition, the levels of inflammatory cytokines TNF-α, IL-6 and MCP-1 were significantly reduced after administration of SZP-L and SZP-H.

### SZP downregulated the expressions of inflammatory cytokines in HG-treated HK-2 cells

**Fig. 5:**
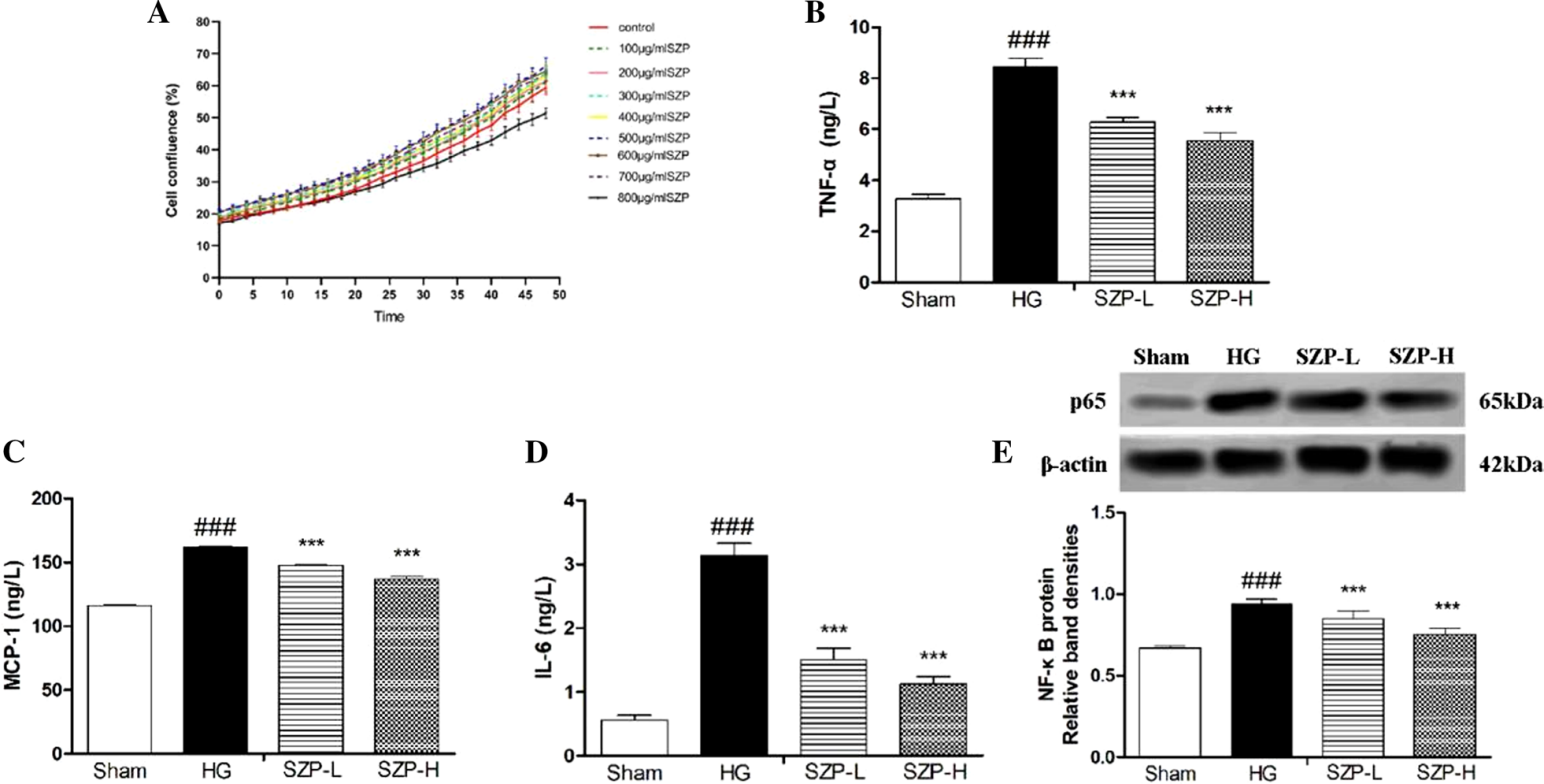
The effects of SZP on the secretion of inflammatory cytokines and proteins in HG-treated HK-2 cells. The cell viability of HK-2 cells after SZP treatment (**a**). The expression level of TNF-α (**b**). The expression level of MCP-1 (**c**). The expression level of IL-6 (**d**). NF-κB protein expressions in HG-stimulate HK-2 cells after SZP treatment (**e)**. The values presented are the means ± SD of three independent experiments (n = 3) and differences between mean values were assessed by ANOVA. ###P < 0.001 versus sham control group, ***P < 0.001 versus HG group. HG, high-glucose; TNF-α, Tumor necrosis factor-α; MCP-1, Monocyte chemotactic protein-1; IL-6, Interleukin 6. NF-κB, Nuclear factor-kappa B

Cell viability and expressions of TNF-α, IL-6, and MCP-1 were analyzed in HG-stimulated HK-2 cells to further study the inhibitory effect of SZP on the production of inflammatory cytokines. SZP-L (100 μg/mL) and SZP-H (200 μg/mL) did not have cytotoxic effects in HK-2 cells as shown in Fig. 5a. These results indicate that SZP could effectively reduce the levels of TNF-α, IL-6, and MCP-1 in HG-treated HK-2 cells. Compared with HG-stimulated HK-2 cells, the high expression levels of TNF-α, IL-6, and MCP-1 were markedly prevented by a 48-h administration of SZP, especially at a dose of 200 μg/mL (Fig. 5b–d).

### SZP inhibited NF-κB activation

NF-κB is involved in the pathogenesis of various diseases (e.g., inflammatory response, carcinogenesis, and apoptosis). NF-κB is an important transcriptional regulator that mediates inflammatory responses. The translocation of NF-κB p65 was analyzed in STZ-induced mice and HG-stimulated HK-2 cells to determine whether SZP inhibits inflammatory cytokine expressions by inhibiting NF-κB activation. Western blotting showed that the NF-κB p65 expression was significantly increased after STZ and HG stimulation. After SZP intragastric administration, NF-κB p65 in the kidney tissue of DN mice was significantly reduced (Fig. 4d). Furthermore, SZP treatment also significantly reduced the high expression of NF-κB p65 in HK-2 cells induced by HG (Fig. 5e).

### Effect of SZP on NF-κB pathway in LPS + TNF-α stimulates HK-2 cells

**Fig. 6:**
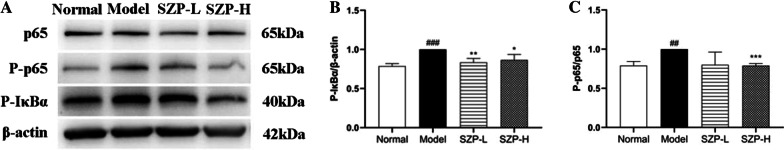
The effects of SZP on Western blot analysis of NF-κB p65 and IκB protein expression in LPS + TNFα stimulate HK-2 cells. **a** Representative blots for NF-κB p65 and IκB protein expression. **b** Bar graph showing IκB protein expression in HK-2 cells treated with LPS and TNFα, **c** Bar graph showing NF-κB p65 protein expression in HK-2 cells treated with LPS and TNFα; The values presented are the means ± SD of three independent experiments (n = 3) and differences between mean values were assessed by ANOVA. ###P < 0.001, ##P < 0.01 and #P < 0.05 versus normal control group, ***P < 0.001,**P < 0.01 and *P < 0.05 versus model group. TNF-α, Tumor necrosis factor-α; NF-κB, Nuclear factor-kappa B. LPS, lipopolysaccharide

The expression levels of NF-κB and IκBα proteins were detected by western blotting to investigate the role of the NF-κB pathway in SZP-treated HK-2 cells. The LPS and TNF-α bind to relevant receptors in HK-2 cells to induce phosphorylation of IκBα and P65, which in turn promotes the activation of NFκB signaling pathway.Western blot results showed that LPS + TNF-α treatment upregulated the phosphorylation levels of NF-κB p65 and IκB proteins in HK-2 cells, whereas SZP decreased the expression of the corresponding proteins (Fig. 6). These results also suggested that SZP possessed a protective effect on the LPS + TNF-α pathway that stimulates HK-2 cells by inhibiting NF-κB.

## Discussion

Growing evidence that the inflammatory processes are critical contributors to DN [18] exists. The current study observed a marked increase in inflammation levels. They were similarly impacted in both STZ-induced mice and HG-treated HK-2 cells. The upregulation of TNF-α, IL-6, and MCP-1 in kidney tissues further supports a pathogenic role in DN progression [19]. In support of these results, this study used in vitro and in vivo models and found that SZP attenuated palmitate-induced kidney damage and downregulation of inflammatory cytokines.

SZP was obtained using the hot-water extraction and alcohol precipitation method, and the physicochemical properties and activities were analyzed. SZP exhibited an obvious anti-inflammatory effect in vivo and in vitro. These results could be due to the monosaccharide composition. Previous studies have demonstrated that glucans and mannans could reduce immunological activity [20,21]. Table 2 shows that the contents of glucose and mannose were high. According to reports, the composition of monosaccharides could be an essential factor in immunological activities [15].

DN was characterized by albuminuria, hyperglycemia, and renal injury. Moreover, inflammation is the pathogenesis of DN [5]. HFD plus STZ-induced mice could develop hyperglycemia, kidney injury, and an immune response throughout the study period. Therefore, this study proposed that the accumulation of albumin and blood glucose leads to kidney injury and inflammation. A close correlation between inflammatory cytokine overload and DN is supported by the findings that DN animal models and patients had a marked capacity for the expression of inflammatory cytokines. Therefore, this study believes that inflammation may play an important role in kidney injury.

Normally, NF-κB is a transcription factor. It could promote transcription of genes encoding proinflammatory cytokines and implicate in the pathogenesis of inflammation-related DN [22,23]. Moreover, NF-κB is a critical factor in the regulation of immune and inflammatory responses by adjusting the expression of inflammatory cytokines. Therefore, the suppression of NF-κB activation is a very effective strategy to prevent inflammatory cytokine production in DN [24]. Thus, examining the possible molecular mechanism of SZP would be interesting. Moreover, the activation of NF-κB signaling was estimated. Strong NF-κB p65 was observed in kidney tissues of STZ-induced mice compared with the sham control group [25,26]. Notably, the number of NF-κB p65 was reduced by SZP (Figs. 4d, 5e). Multiple pieces of evidence indicated that the levels of TNF-α were crucial for the activation of NF-κB [27]. Consequently, investigators proposed that TNF-α may promote NF-κB transcription. Therefore, it is hypothesized that TNF-α may be a mediator for SZP to inhibit the NF-κB signaling pathway [28,29]. The results showed that both TNF-α and NF-κB were increased in DN conditions (in DN mice and high-glucose-treated HK-2 cells) and reversed by SZP treatment (Figs. 4, 5). The NF-κB expression when LPS + TNF-α was stimulated in HK-2 cells was next detected to test whether NF-κB pathway activation involved in the anti-inflammatory effects of SZP is mediated by TNF-α. As expected, the inhibitory effect of SZP on NF-κB pathway activity was markedly by inhibiting the phosphorylation of p65 and IκBα (Fig. 6).

As an important role in DN, NF-κB may have various biological functions. For example, NF-κB has been shown to possess anti-inflammatory effects by increasing inflammation in the early stage of DN [30,31]. In the current study, the protein expression of NF-κB and the proinflammatory mediators IL-6 and MCP-1 in the supernatant of HG-treated HK-2 cells were significantly upregulated following treatment with SZP compared with those in the sham control group. It suggests that NF-κB may act as a mediator linking tubular cell injury to interstitial IL-6 and MCP-1 in DN. The pathogenic role of NF-κB in linking DN has also been supported by recent reports showing that IL-6 and MCP-1 overexpression was associated with the activation and inhibition of the NF-κB pathway. Furthermore, the levels of NF-κB protein and the secretion of IL-6 and MCP-1 in the DN group were all higher compared with the sham control group. These results were consistent with the findings of the previous studies [32–35].

This work supports the hypothesis that SZP is a protective factor in the kidney. Moreover, SZP may be an important treatment strategy in diabetic nephropathy.

## Conclusions

In conclusion, the study preliminarily demonstrates that SZP has a strong renal protective effect on type II diabetic nephropathy, which are related to the improvement of blood sugar, lipid metabolism, and kidney inflammation. Moreover, the underlying mechanism may be that SZP can alleviate diabetic kidney damage by inhibiting inflammation via NF-κB pathway.

## Data Availability

The datasets used or analysed during the current study are available from the corresponding author on reasonable request.

## Abbreviations

DN: Diabetic nephropathy
T2DM: Type 2 diabetes mellitus
NF-κB: Nuclear factor-kappa B
MET: Metformin
SZGB: Sanziguben
SZP: Sanziguben polysaccharides
HK-2: Human tubule epithelial cells
HFD: High-fat diet
STZ: Streptozotocin
HG: High glucose
ELISA: Enzyme-linked immunosorbent assay
TNF-α: Tumor necrosis factor-alpha
LPS: Lipopolysaccharides
P-p65: Phospho-NF-κB p65
P-IκBα: Phospho-IκBα
RIPA: Radioimmunoprecipitation assay lysis buffer
BCA: Bicinchoninic acid
HRP: Horseradish peroxidase
FBG: Fasting blood glucose
GC: Gas chromatography
FTIR: Fourier transform infrared spectroscopy
TC: Total cholesterol
TG: Triglycerides
Cr: Serum creatinine
BUN: Serum urea
MDA: Malondialdehyde
CAT: Catalase
MCP-1: Monocyte chemotactic protein-1
IL-6: Interleukin 6
H&E staining: Stained with hematoxylin & eosin
PAS: Periodic acid-Schiff
SDS-PAGE: Sulfate–polyacrylamide gel electrophoresis
PVDF: Polyvinylidene difluoride
